# Synergic Interactions Between Hepatic Stellate Cells and Uveal Melanoma in Metastatic Growth

**DOI:** 10.3390/cancers11081043

**Published:** 2019-07-24

**Authors:** Léo Piquet, Louise Dewit, Nathan Schoonjans, Martial Millet, Julie Bérubé, Peter R. A. Gerges, François Bordeleau, Solange Landreville

**Affiliations:** 1 Faculté de médecine, Université Laval, Quebec City, QC G1V 0A6, Canada; 2 Centre de recherche du CHU de Québec-Université Laval, Quebec City, QC G1S 4L8, Canada; 3 Centre de recherche sur le cancer de l’Université Laval, Quebec City, QC G1R 3S3, Canada; 4 Centre de recherche en organogénèse expérimentale de l’Université Laval/LOEX, Quebec City, QC G1J 1Z4, Canada

**Keywords:** metastatic uveal melanoma, hepatic stellate cells, xenografts, fibrogenesis, histopathological growth patterns, quantitative polarization microscopy

## Abstract

Uveal melanoma (UM) is a malignant intraocular tumor that spreads to the liver in half of the cases. Since hepatic cells could play a role in the therapeutic resistance of metastatic UM, the purpose of our study was to investigate the pro-invasive role of hepatic stellate cells (HSteCs) in metastatic UM at the micro- and macro-metastatic stages. We first performed an immunostaining with the alpha-smooth muscle actin (αSMA) to localize activated HSteCs in UM liver macro-metastases from four patients. Their accumulation of collagen was assessed with Masson’s Trichrome stain. Next, we inoculated metastatic UM cells alone or with human HSteCs in triple-immunodeficient mice, in order to determine if HSteCs are recruited as early as the micro-metastatic stage. The growth of metastatic foci was imaged in the liver by *ex vivo* fluorescence imaging. Histological analyses were performed with Masson’s Trichrome and Picrosirius Red stains, and antibodies against Melan-A and αSMA. The collagen content was measured in xenografts by quantitative polarization microscopy. In patient hepatectomy samples, activated HSteCs and their pathological matrix were localized surrounding the malignant lesions. In the mouse xenograft model, the number of hepatic metastases was increased when human HSteCs were co-inoculated. Histological analyses revealed a significant recruitment of HSteCs near the micro/macrolesions, and an increase in fibrillar collagen production. Our results show that HSteCs can provide a permissive microenvironment and might increase the therapeutic resistance of metastatic UM.

## 1. Introduction

Uveal melanoma (UM) is the most common primary intraocular tumor in adults [1], and is biologically distinct from skin melanoma [2]. It is a sporadic tumor that arises from the malignant transformation of neural crest-derived melanocytes found in the uveal tract, with a higher occurrence in the choroid [1]. Radiation therapy or surgical eye removal have allowed for satisfactory local tumor control [3,4], but half of the patients develop metastases, mainly in the liver and the survival rate at this stage is less than 10% at 2 years [1,5,6]. Powerful prognostic tools help to stratify UM cases according to their risk of liver metastasis [7,8,9,10,11,12]. However, the "Achilles' heel" of metastatic UM has yet to be found. Although immunotherapy is curative in subsets of patients and has dramatically changed the treatment approach to skin melanoma [13,14], only rare complete responses with adoptive T-cell therapy or immune checkpoint blockade have been reported in metastatic UM [15,16,17]. Moving forward with treatments to control/cure the metastatic stage of this cancer will require a better understanding of mechanisms that allow the stromal cells such as hepatic stellate cells (HSteCs) to create a permissive environment. In their quiescent state in healthy liver, HSteCs are vitamin A-storing cells present in the sub-endothelial space of Disse; they account for 5–8% of the hepatic cells [18]. Activated HSteCs, which are proliferative and inflammatory, play pivotal roles in liver disease pathogenesis [18]. They are involved among others in the metastatic progression of colorectal and pancreatic cancers, in which they transdifferentiate into contractile extracellular matrix-producing myofibroblasts positive for the alpha-smooth muscle actin (αSMA) [18,19,20,21]. Their fibrogenesis activity may be detrimental to the uptake of anti-cancer drugs in liver metastases [22]. Activated HSteCs were previously spotted surrounding UM metastases [23,24], and we recently showed that the bidirectional crosstalk between metastatic UM cells and HSteCs involved pro-fibrogenic interleukins [25]. The purpose of our study was thus to investigate the pro-invasive role of HSteCs in metastatic UM at the micro- and macro-metastatic stages. The metastatic load and the recruitment of activated HSteCs were determined in immunodeficient mice after the co-inoculation of human HSteCs with metastatic UM cells. In addition, the production of fibrillar collagen by HSteCs in the vicinity of malignant lesions was studied in xenografts and UM patients’ samples.

## 2. Results

### 2.1. Presence of Activated HSteCs in UM Patients’ Hepatectomy Samples

**Figure 1:**
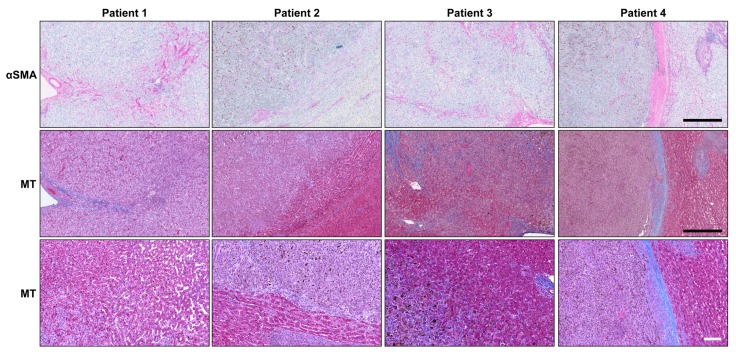
Activated HSteCs are abundant in UM patients’ liver macro-metastases. Immunohistological analyses of UM patients’ macro-metastases (*N* = 4) using the αSMA antibody to label activated stellate cells; their positive signal appears in magenta (top panels). The accumulation of collagen produced by activated HSteCs is visible in blue in the vicinity of malignant lesions (MT; middle panels). The higher magnification of the invasive margin allows to determine the HGP for each metastasis (bottom panels). Black scale bars, 500 µm; white scale bar, 100 µm.

Considering that activated HSteCs play a pivotal role in the metastatic growth of other tumors with a hepatic tropism, we first investigated their localization in liver macro-metastases resected from UM patients (Figure 1). Using αSMA as a marker for activated HSteCs during pathogenesis [26], we observed that activated HSteCs were indeed abundant near the UM malignant lesions (Figure 1). 

**Figure 2:**
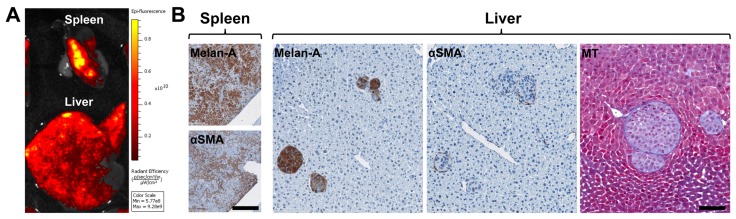
Establishment of a mouse xenograft model of metastatic UM to assess the recruitment of HSteCs. (**A**) Ex vivo fluorescence imaging of the liver and spleen of a triple-immunodeficient mouse bearing UM metastases generated with the TJU-UM001 cell line co-inoculated with human HSteCs. The color scale indicates the intensity of the fluorescence recorded 6 weeks post-inoculation, where the yellow signal corresponds to the highest concentration of UM cells. (**B**) Immunohistological analyses of UM-derived liver metastases using the Melan-A antibody to identify melanoma malignant lesions, and the αSMA antibody to label activated stellate cells; the positive signal appears in brown. The Masson’s Trichrome (MT) stain reveals the presence of collagen in blue. Scale bars, 100 µm.

An accumulation of collagen was seen around these αSMA-positive cells, as highlighted by the Masson’s Trichrome stain (MT; Figure 2). In Patient 4, activated HSteCs even accumulated a thick ring of extracellular matrix around the macro-metastasis, a defining characteristic of the desmoplastic histopathological growth pattern (HGP) [27]. Since our human liver metastasis samples did not include micro-metastases, we developed a mouse xenograft model of metastatic UM to determine if HSteCs are recruited early when metastatic UM cells invade the liver.

### 2.2. A Mouse Xenograft Model to Study the Recruitment of Stellate Cells by Metastatic UM

**Figure A1:**
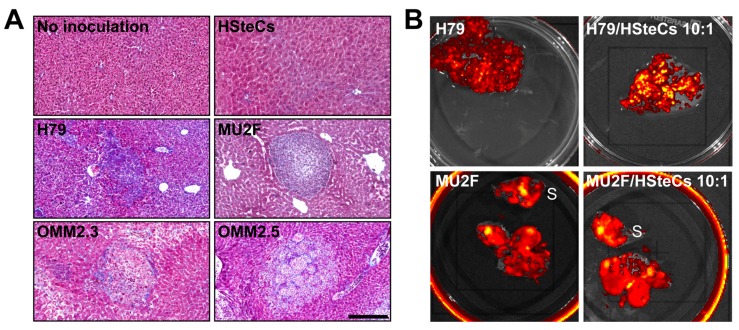
HSteCs are activated by various metastatic UM cell lines. (**A**) The Masson’s Trichrome (MT) stain reveals the production of collagen by HSteCs (in blue) in the vicinity of malignant lesions generated in triple-immunodeficient mice using metastatic UM cell lines H79, MU2F, OMM2.3 and OMM2.5. There is no malignant lesion in control groups without inoculation of human cells or inoculated with human HSteCs alone. Scale bar, 50 µm. (**B**) *Ex vivo* fluorescence imaging of the liver of triple-immunodeficient mice bearing UM metastases generated with H79 and MU2F cell lines inoculated alone or in combination with human HSteCs (ratio 10:1). S = spleen. The red to yellow signal corresponds to malignant lesions at 6 weeks post-inoculation.

We established a mouse xenograft model of metastatic UM by injecting metastatic UM cells with human HSteCs into the spleen of triple-immunodeficient mice (Table 2; Figure 2 and Appendix A Figure A1). Using an in vivo imaging system (IVIS), we confirmed that the metastatic UM cell line TJU-UM001 was correctly injected into the spleen, and developed hepatic metastases in 6 weeks (Figure 2A). We then performed histological analyses to localize UM cells and activated HSteCs in both organs (Figure 2B), as well as to determine the predominant HGP in the xenografts (Table 2). Interestingly, the predominant HGP was different between UM cell lines, suggesting that their invasive growth properties were conserved despite their in vitro culture. The Melan-A positive staining in the spleen demonstrated the implantation of melanoma cells; co-injected HSteCs were also detected using αSMA (Figure 2B, left panels). The number of UM cells inoculated in mice was optimal since hepatic malignant lesions that varied in sizes were generated in 6 weeks, representative of both micro-metastatic and macro-metastatic stages (Figure 2B, right panels). In addition, the αSMA positive staining highlighted HSteCs surrounding all metastases, while the Masson’s Trichrome (MT) blue stain clearly demonstrated the presence of newly synthetized extracellular matrix around UM malignant lesions (Figure 2B, right panels). We did not observe any difference in the metastatic load between NOD CRISPR *Prkdc Il2r Gamma* (NCG) and NOD *scid Gamma* (NSG) triple-immunodeficient mice or when the hTERT-HSC cell line versus primary HSteCs were co-injected with UM cells. 

### 2.3. Increase in the Number of UM Hepatic Metastases when Human HSteCs Are Co-inoculated

**Figure 3:**
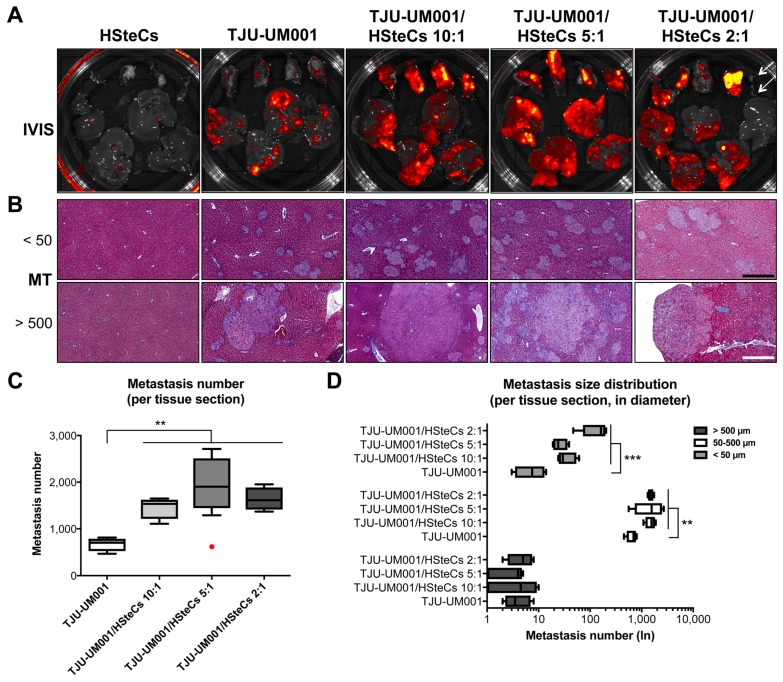
HSteCs interact with TJU-UM001 cells to favor UM liver metastasis and extracellular matrix production. (**A**) Ex vivo fluorescence imaging (IVIS) of the spleen (top part of the Petri dish) and liver (bottom part of the Petri dish) of triple-immunodeficient mice (*N* = 4 per condition) bearing UM metastases generated with the TJU-UM001 cell line inoculated alone or in combination with human HSteCs (ratios 10:1, 5:1 or 2:1). The red to yellow signal corresponds to malignant lesions at 6 weeks post-inoculation. Arrows on the last panel indicate the spleen and liver of a mouse that was not inoculated with human cells. (**B**) Masson’s Trichrome (MT) stain reveals the production of collagen (in blue) in the vicinity of UM micro-metastases (<50 µm) and macro-metastases (>500 µm). Black scale bar, 50 µm; white scale bar, 500 µm. (**C**) The metastasis number for the four groups is represented as box-and-whisker plots, with the median indicated by the horizontal line. The red dot corresponds to an outlier. One-way ANOVA with Dunnett’s post hoc test, * *p* < 0.05 and ** *p* < 0.01. (**D**) The metastasis size distribution in diameter (<50 µm, 50–500 µm or >500 µm) for the four groups is represented as box-and-whisker plots, with the median indicated by the horizontal line. One-way ANOVA with Dunnett’s post hoc test, * *p* < 0.05, ** *p* < 0.01 and *** *p* < 0.001.

**Figure 4:**
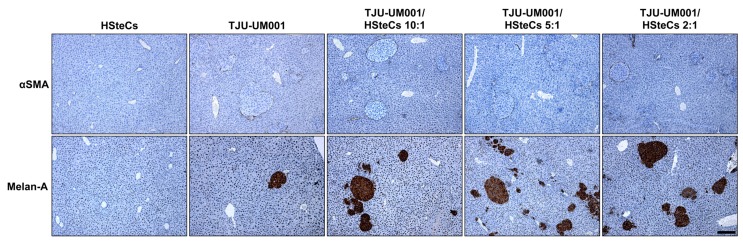
HSteCs are recruited early by metastatic UM cells. Immunohistological analyses of UM-derived liver metastases in triple-immunodeficient mice (*N* = 4 per condition) inoculated with the TJU-UM001 cell line alone or in combination with human HSteCs (ratios 10:1, 5:1 or 2:1). The αSMA antibody labels activated stellate cells recruited in the vicinity of UM cells, while the Melan-A antibody highlights UM malignant lesions; the positive signal appears in brown. Scale bar, 100 µm.

As the main objective of our study was to assess the synergic interactions between UM cells and HSteCs in metastatic growth, we then co-inoculated the TJU-UM001 cell line with different ratios of human HSteCs in triple-immunodeficient mice (Figure 3 and Figure 4). Fluorescent HSteCs inoculated alone migrated to the liver without forming lesions as shown in Figure 3A. The metastatic load increased in mice co-inoculated with 10:1, 5:1 or 2:1 ratios of HSteCs compared to TJU-UM001 cells injected alone (Figure 3A). Similar results were obtained when H79 and MU2F cell lines were co-injected with HSteCs at a ratio of 10:1 (Table 2, Appendix A Figure A1B).

Activated HSteCs were recruited by both micro- (<50 µm, top panels) and macro-metastases (>500 µm, bottom panels; Figure 3B and Figure 4). They produced fibrillar collagen in the vicinity of UM malignant lesions in all co-inoculation groups similarly to human samples (Figure 1), as well as in mice injected only with the TJU-UM001 cells (Figure 3B and Figure 4). In the latter instance, αSMA-positive cells surrounding UM metastases most likely correspond to endogenous murine HSteCs. Next, hepatic metastases were localized and count in all groups using MT-stained slides (Figure 3C). The co-inoculation of TJU-UM001 with human HSteCs led to an increase in the number of hepatic metastases regardless of the ratio (*p* = 0.0033).

We also analyzed the size in diameter of the metastases, to see if the ratios of HSteCs impacted their distribution between micro-and macro-metastases (Figure 3D). Once again, the co-inoculation of TJU-UM001 cells with human HSteCs increased significantly the number of micro-metastases (<50 µm; *p* = 0.0007) and metastases between 50–500 µm (*p* = 0.0054), even at the 10:1 ratio. These results confirmed the early role of stellate cells in favoring the growth and invasion of UM cells in the liver.

### 2.4. Increase of Collagen Content when Human HSteCs Are Co-inoculated

**Figure 5:**
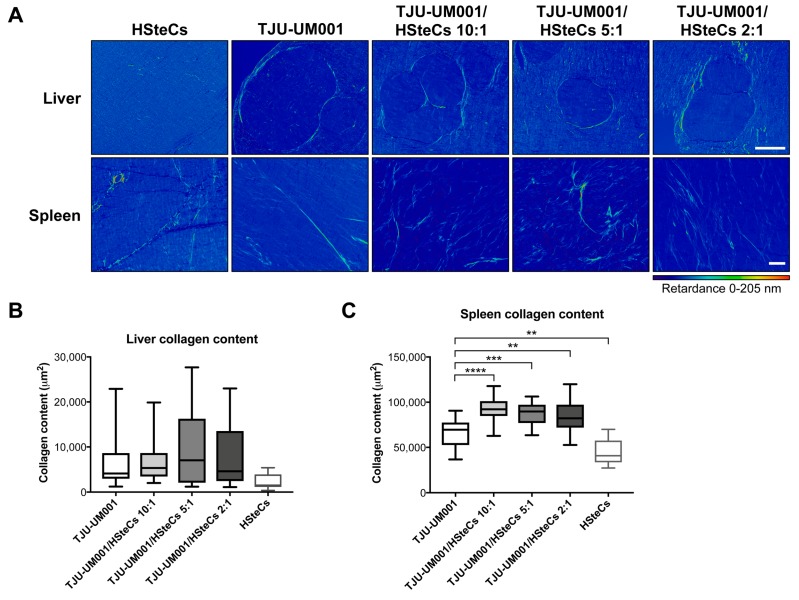
Human HSteCs accumulate fibrillar collagen when co-injected with metastatic UM cells. (**A**) QPOL imaging of the liver (top panels) and spleen (bottom panels) of triple-immunodeficient mice (*N* = 4 per condition) bearing UM metastases generated with the TJU-UM001 cell line inoculated alone or in combination with human HSteCs (ratios 10:1, 5:1 or 2:1). The dark blue to red signal corresponds to the collagen content (retardance scale) at 6 weeks post-inoculation. Scale bars, 100 µm. The collagen content in the liver (**B**) or spleen (**C**) expressed in µm^2^ for the five groups is represented as box-and-whisker plots, with the median indicated by the horizontal line. One-way ANOVA with Dunnett’s post hoc test, ** *p* < 0.01, *** *p* < 0.001 and **** *p* < 0.0001.

Given that activated HSteCs were recruited in the vicinity of metastases of all sizes, and that collagen deposition was observed with the Masson’s Trichrome stain, we characterized in more detail the collagen content within the metastatic microenvironment using quantitative polarization (QPOL) microscopy (Figure 5). Surprisingly, imaging of Picrosirius Red-stained collagens I and IV within the samples indicated that the collagen architecture and content were similar between all groups in the liver (Figure 5A top panels and B; *p* = 0.0512). Since the host’s HSteCs can also contribute to the collagen deposition near the malignant lesions, we investigated the collagen micro-architecture into the spleen, where there are no murine stellate cells. In this case, all spleens injected with both human HSteCs and UM cells showed a significant increase in collagen content and presented regions of altered fibrillar collagen architecture compared with the spleens injected only with UM cells (Figure 5A bottom panels and C; *p* < 0.0001). 

Overall, our data demonstrate that HSteCs are activated by UM cells as early as the micro-metastatic stage, and their synergic interactions lead to modifications of the hepatic stroma and an increase of the metastatic growth.

## 3. Discussion

In recent years, components of the microenvironment of solid tumors, such as stromal cells and their pathological extracellular matrix, have gained attention because they can significantly impair the therapeutic response. Our findings support the hypothesis that HSteCs are the most reactive stromal cells of the liver in presence of invading UM cells. Indeed, we showed that matrix-producing stellate cells are abundantly present in UM patients’ hepatic macro-metastases. We thus established a mouse xenograft model of metastatic UM to assess if stellate cells were involved early in the growth of UM cells in the liver. We demonstrated that activated stellate cells were recruited at both the micro- and macro-metastatic stages, and they synthesized fibrillar collagen in the vicinity of metastases. In addition, the metastatic load was increased in immunodeficient mice when human HSteCs were co-injected, confirming that their synergetic interactions increased the liver susceptibility to UM invasion.

Coupland and collaborators previously identified “hepatic fibrosis” and “HSteC activation” among the most differentially regulated biological processes when they compared secretomes of UM cells and choroidal melanocytes by proteomic profiling [28]. In addition, the paracrine signaling of HSteCs potentiates UM aggressiveness in vitro [25,29]. Indeed, Aplin and collaborators demonstrated that the HSteC conditioned medium, that contains among others the hepatocyte growth factor, increased the migration and invasion of UM cells, as well as their resistance to a MEK inhibitor [29]. We previously found that metastatic UM cells were more responsive to the secretome of HSteCs than non-metastatic cells, which generated a pro-angiogenic and pro-inflammatory microenvironment, with no effect on UM cell proliferation [25]. Our mouse xenograft model of metastatic UM allowed to confirm the early recruitment of HSteCs by UM micro-metastases. Co-inoculated human HSteCs created a permissive microenvironment that led to the homing of more hepatic lesions. Importantly, we also observed that the xenografts had defining characteristics of desmoplastic or replacement HGPs [27]. Barnhill and collaborators previously determined that the replacement pattern was correlated to shorter overall survival of UM patients [30]. 

We previously demonstrated a higher secretion of the pro-fibrogenic interleukins IL-6 and IL-8, and an increased expression of transmembrane integrins when metastatic UM cells were co-cultured with HSteCs [25]. Our histological analyses showed that αSMA-positive stellate cells co-localized with newly synthetized extracellular matrix, particularly fibrillar collagen, in both human and xenografted hepatic metastases. In addition, we confirmed an increased fibrogenesis from human HSteCs in contact with UM cells by studying the splenic injection site, which did not contain murine stellate cells. Burnier and collaborators previously revealed that the collagen IV was abundant in human hepatic metastases from several types of tumors [31]. Further analyses of the composition and stiffness of the pathological extracellular matrix produced by activated HSteCs in UM micro- and macro-metastases will thus be required. Increasing evidence implicates the extracellular matrix stiffness in cancer progression [32,33,34,35]. Interestingly, patients with a stiffer fibrotic liver have higher metastatic incidence and lower survival rate [34]. Since cells react to the remodeling of the extracellular matrix by sensing changes in mechanical properties [36,37], mechanotransduction pathways have recently gained attention as therapeutic targets in cancer, including in UM [38,39]. Our mouse xenograft model humanized with HSteCs will be of great interest to test such mechano-based therapeutic interventions in metastatic UM [38].

## 4. Materials and Methods

### 4.1. Human Metastatic Tissues and Cell Lines

Liver metastasis tissues were resected from UM patients, after their written informed consent was obtained (Table 1; RRCancer-CRCHUM Biobank, Université de Montréal). Metastatic UM cell lines TJU-UM001, H79, MU2F, OMM2.3 and OMM2.5 were derived from liver metastases and expended as previously described [40,41,42,43]. Human primary hepatic stellate cells (HSteCs; ScienceCell Research Laboratories, Carlsbad, CA, USA) and the hTERT-HSC cell line were grown as previously described [44], and co-injected with UM cells in immunodeficient mice. All cells were grown at 37 °C in a humidified atmosphere with 5% CO_2_. This study followed the tenets of the Declaration of Helsinki and was approved by our institutional human experimentation committee (Centre de recherche du CHU de Québec-Université Laval, protocol #2012-1483).

### 4.2. Mouse Xenograft Model

One million of fluorescent UM cells (tdTomato or Green Fluorescent Protein) were inoculated alone or with HSteCs (100,000–500,000 cells; stained with DiI or DiO) into the spleen of NOD CRISPR *Prkdc Il2r Gamma* (NCG; Charles River Laboratories, Sherbrooke, QC, Canada) or NOD *scid Gamma* (NSG; The Jackson Laboratory, Bar Harbor, ME, USA) triple-immunodeficient mice (Table 2). Six weeks after the inoculation, mice were euthanized, and the growth of malignant lesions was assessed by fluorescence in harvested liver and spleen using an IVIS (Perkin Elmer, Woodbridge, ON, Canada). Both organs were then fixed with 2% paraformaldehyde and embedded in paraffin for histological analyses. All animal experiments were conducted in voluntary compliance with the ARVO Statement for the Use of Animals in Ophthalmic and Vision Research, and were approved by our institutional animal experimentation committee (Comité de protection des animaux de l’Université Laval, protocol #17-016).

### 4.3. Histological Analysis and Liver Metastasis Quantification

Five-µm liver sections were colored with the Masson’s Trichrome (MT) stain. Tissue sections were also processed for immunohistological analysis with antibodies against Melan-A (clone A103, Agilent Dako, Mississauga, ON, Canada) and αSMA (clone 1A4, Agilent Dako) in a Dako Autostainer Plus Link, according to the manufacturer’s protocol using the EnVision peroxidase procedure with the DAB or Magenta chromogen (Agilent Dako) [45]. The demasking was done at high pH. Secondary antibody without primary antibody was used as negative control, and positive controls were performed with skin and muscle tissues. Sections were digitized with a slide scanner (NanoZoomer 2.0HT; Hamamatsu Photonics, Bridgewater, NJ, USA), and images were analyzed with the NDP.view 2 software (Hamamatsu Photonics). Liver malignant lesions were counted on MT-stained slides using the measure feature of NDP.view 2. They were then categorized according to their size (<50 µm, 50–500 µm, >500 µm in diameter) [23]. 

### 4.4. Quantitative Polarization (QPOL) Microscopy

Liver sections were colored with the Picrosirius Red stain. The polarized signal was then imaged and quantified using both white and monochromatic red lights in QPOL as described previously [46,47]. Briefly, QPOL and colorimetric imaging were performed using an Axio Vert microscope (Zeiss, Toronto, ON, Canada) equipped with a 10× 0.35 N.A. and 20× 0.5 N.A. polarization objectives, and Axiocam 305 monochromatic and 105 color cameras (Zeiss). The microscope is composed of a motorized rotating linear polarizer (max speed of 20° s^−1^; Thorlabs, Newton, NJ, USA) positioned directly under the illumination source and above the condenser and a circular analyzer [47]. The Zen lite software was used for image acquisition (Zeiss). For QPOL, an image sequence was acquired using the Axiocam 305 at each 10 step of the rotating polarizer over a range from 0° to 180°. The sequence of images was then processed with a MATLAB code in order to obtain a pixel-by-pixel retardance image, from which the area of the collagen regions was extracted and analyzed using the ImageJ software (http://rsb.info.nih.gov/ij/). For colorimetric image analysis, acquisitions were done using the Axiocam 105 and the Picrosirius Red signal was isolated from the resulting RGB image by subtracting the green and blue channels from the red channel. A threshold was then applied to the computed image using the Otsu’s method and the total area of collagen regions was calculated.

### 4.5. Statistical Analysis

Data are presented as box-and-whisker plots. The central box is the interquartile range with the median indicated by the horizontal line, and whiskers extend to the lowest and highest data values. Data were analyzed for statistical significance (*p* < 0.05) using one-way ANOVA followed by Dunnett’s multiple comparisons post hoc test (Prism 7, GraphPad Software, San Diego, CA, USA).

## 5. Conclusions

We demonstrated that the co-inoculation of metastatic UM cells with human HSteCs increased the number of metastatic lesions smaller than 500 µm in our experimental model of UM liver metastasis. An accumulation of pathological extracellular matrix was co-localized with the activated αSMA-positive stellate cells in the vicinity of metastases. This matrix remodeling and contraction may increase the intratumoral interstitial fluid pressure, which will be detrimental for the uptake of anticancer drugs. New combined therapies targeting both the metastatic UM cells and the fibrogenesis of stellate cells are thus warranted.

## Figures and Tables

**Table 1:** Metastatic uveal melanoma patients’ survival data.

Patient	Sex/Age ^1^	Initial Diagnosis	Liver Surgery	Histopathological Growth Pattern ^2^	Follow-Up ^3^ (month)	Treatment of Metastases before Resection	Last Status ^4^
1	F/67	2009	12/2012	replacement	18	No	DOM
2	F/53	2005	01/2014	mixed	111	No	AWM
3	F/69	2013	12/2015	replacement	50	No	AWM
4	F/53	1990	03/2018	desmoplastic	14	Immunotherapy	AWM

^1^ Age at the initial diagnosis of the ocular tumor. ^2^ HGP: scored according to international guidelines [27]. ^3^ Follow-up: period from liver surgery until patient death or last visit. ^4^ Last status: DOM, dead of metastasis; AWM, alive with metastasis.

**Table 2:** Summary of liver metastasis formation in immunodeficient mice inoculated with human UM cell lines and HSteCs.

Cell Lines	Mice with Metastasis	Histopathological Growth Pattern
HSteCs	0/20	-
TJU-UM001	17/17	desmoplastic
TJU-UM001 + HSteCs	30/30	desmoplastic
H79	3/3	mixed
H79 + HSteCs	3/3	mixed
MU2F	6/7	replacement
MU2F + HSteCs	3/3	replacement
OMM2.3	4/4	replacement
OMM2.5	4/4	desmoplastic
